# Patients Characteristics and Preliminary Outcomes of Heart Failure Registry in A Middle-Income Country: Persian Registry of Cardiovascular Disease/Heart Failure (PROVE/HF)

**DOI:** 10.22086/gmj.v0i0.1026

**Published:** 2018-11-08

**Authors:** Mahshid Givi, Davood Shafie, Mohammad Garakyaraghi, Ghasem Yadegarfar, Hamid Reza Roohafza, Seyed Abdollah Ahmadi, Fatemeh Nouri, Nizal Sarrafzadegan

**Affiliations:** ^1^Heart Failure Research Center, Cardiovascular Research Institute, Isfahan University of Medical Sciences, Isfahan, Iran; ^2^Isfahan Cardiovascular Research Center, Cardiovascular Research Institute, Isfahan University of Medical Sciences, Isfahan, Iran; ^3^Department of Biostatistics and Epidemiology, School of Public Health, Isfahan University of Medical Sciences, Isfahan, Iran

**Keywords:** Heart Failure, Registries, Disease Management, Iran

## Abstract

**Background:**

The Persian Registry of Cardiovascular disease/Heart Failure (PROVE/HF) aimed to studied the demographic, clinical, and diagnostic characteristics and treatment of patients hospitalized for heart failure (HF) and to follow them for short- and long-term outcomes. Its pilot phase started in 2015 in Isfahan aiming to evaluate its feasibility to be scaled up at the national level in later stages. This article describes the method and preliminary results of the first year registry.

**Materials and Methods:**

Information of hospitalized patients with preserved and low ejection fraction, were gathered. Patients were followed for 1, 6, and 12 months. During follow-up, information of the patients’ current status, medications used during hospitalization, and in case of death, the cause and place were assessed.

**Result:**

PROVE/ HF enrolled 787 patients in the first year. The mean age of patients was 70.74 ±12.01 years, and 60.7% of them were men. The most frequent risk factors for the development of HF in the recruited patients was ischemic heart disease (77.9%), and hypertension (63.7%), respectively. The re-admission rate for patients with HF was at least once in 16% and continued until the fifth to ninth re-admission over a one-year period. Among 787 registered patients, 30.9% died in the first year of follow-up, and the in-hospital mortality was 6.2%. The mean hospitalization period was 4.88 days, and 64.2% were hospitalized for >3 days.

**Conclusion:**

The annual rate of re-admission and mortality was high, and the use of medication was less than the recommended one inaccordance with the guidelines for the treatment of heart failure.

## Introduction

A gradual decrease in mortality rates, in line with increased life expectancy, has increased the elderly population of the world [1]. Iran is not exempt from this change. Currently, the age pyramid of Iranian population is changing from youth to elderly [2]. Age increase leads to the decreased health of the cardiovascular system [3]. Several cross-sectional and cohort studies in Isfahan have warned the Iranian population about the prevalence of cardiovascular diseases and its risk factors [4-5]. Among these, heart failure (HF) is the final common path of all cardiovascular diseases [6]. HF is a very serious condition in which the heart cannot pump or fill with enough blood to provide adequate blood flow to other organs [7]. HF is a common lethal, and life-threatening disease [8] with increased prevalence in recent years [9]. The more the population ages, the more HF is considered as an important health problem [10]. Currently, cardiovascular mortality has decreased following new therapies [11], which increases the proportion of patients with HF. In addition to the high mortality rate in patients with HF [12], increased re-admission of patients [13], increases the length of hospital stay, and hospital costs [14]. Moreover, the reduced quality of life of these patients [15] is an important issue associated with HF. Meanwhile, efficient management is necessary for the increased efficiency of health services. However, an efficient information system is the prerequisite for reasonable management [16].

Researchers believe that having a registry system for documenting the information of patients with HF, analysis of obtained data, and comparison with other countries are the prerequisite of improving the quality of healthcare in this field. Thus, the Persian Registry of Cardiovascular Disease/Heart Failure (PROVE/HF) program, as a pilot study in Isfahan and for the first time in Iran, recorded data of patients admitted with HF and followed them. [17]. Why so comprehensive information of clinical management and epidemiology of HF are not available in the Asian region that makes it hard to compare these issues with Western countries [18].

## Materials and Methods

To register information of patients with HF, a researcher-designed questionnaire was used, which was designed according to the results of the Swedish Heart Failure Registry (S-HFR) [19] and Thai Acute Decompensated Heart Failure Registry (Thai ADHERE) [20].

The questionnaire included demographic data, underlying diseases and comorbidities, medications and treatments, diagnostic and laboratory test results, symptoms and signs, and results of examination during hospitalization. To check content validity, after completion, ten faculty members, cardiologists, and experts from outside the project were asked to determine whether the questions measure the desired outcome or not and whether the question include the entire content of what was needed. Since ten experts evaluated the questions, the minimum acceptable content validity ratio (CVR) was considered to be 62%. Questions with CVR value less than this amount were excluded from the questionnaire, and the minimum acceptable value for content validity index (CVI) was considered 0.79, so if CVI of a question was less than 0.79, it would be excluded [21]. After validation of the questionnaire, the protocol and the related dictionary were written, which, in addition to a full description of the procedure, explained all questions, options, and codes. Finally, questionnaires, protocol, and dictionary were approved by quality control (QC) of PROVE Committee.Then, based on the prepared questionnaire, protocol, and dictionary, data entry was taught to the personnel. The training began with three two-hour sessions on how to extract data from medical records, complete data sheets, and reading diagnostic tests as needed, as well as an explanation of the objectives and the protocol. Moreover, monthly training sessions were held, and the team included managers and principal investigators who were allowed to visit the archives of the hospital and correct existing mistakes.

The patients who diagnosed with HF based on International Classification of Diseases, 10h Revision (ICD-11), such as preserved and low ejection fraction (EF), who were hospitalized in 18 private and university hospitals in Isfahan, as well as public, private, military, and charity centers were enrolled in the study. Patients with available medical records in the archives of the hospital who had either acute or exacerbated and decompensated HF were given to data collectors. Diagnosis of HF confirmed by a cardiologist in medical records and included in the medical records. Patients signed the consent form before admission to the hospital. For data confidentiality, personal information of patients was not identified by name, but by national and registry code, a code unique to each individual and was obtained by a formula from patient’s information such as last name, first name, date of birth, prepared from Huffman phonetic codes [22-23].

Data collectors recorded data in questionnaires using the data in the medical records of hospitals’ archives. Then, they delivered the completed questionnaire to the registry unit at the Isfahan Cardiovascular Research Institute (ICRI), a WHO collaborating center on a weekly basis. The questionnaires were reviewed by the data management team and missed, or mistaken cases were returned. Data were entered into the relevant software after being approved by the data management team.

Patients were followed-up after 1, 6, and 12 months by telephone and, if necessary (in cases such as doubt about HF diagnosis or unavailability of basic information in the records) were visited by a specialist. During follow-up, information on the patients’ current status, medications used and in case of death, the cause and place were assessed.PROVE/HF program, in addition to internal QC, was performed by the team’s supervisor, was externally evaluated by the committee, consisting of experienced and trained members who were not one of the PROVE executive members, and were unaware of it and performed an external and continuous control over the entire registry components from the beginning to the end.

### Statistical Analysis

Data were analyzed by SPSS version 22.0 (SPSS Inc., Chicago, IL). Descriptive statistics were calculated based on the clinical characteristics of the registered patients. Quantitative variables were expressed as mean±SD or median and interquartile range (if required). Qualitative variables were summarized as counts (percent). Also for assessing the content validity of the questionnaire, CVR and CVI scores were used. ResultA total of 787 patients with decompensated or acute HF from 18 hospitals in Isfahan were recruited between April 2015 and April 2016.

Table-1 displays the demographic data of patients registered in the first year of registration. The mean age of patients was 70.74±12.01 years, and most of them were men. Among patients with calculated height and weight, the mean body mass index (BMI) was 26.11±4.24. Also, 23.6% of the patients currently smoked or had a history of smoking (21.4% were men, and 2.2% were women).

**Table 1:** Characteristics of Patients Hospitalized with HF

**Characteristics**	**n(%)**
**Age**	70.74 ± 12.01*
**Men**	478 (60.7)
**Medical history and comorbidities**	
**Hypertension**	613 (77.9)
**Ischemic heart disease**	501 (63.7)
**Diabetes mellitus**	371 (47.1)
**Kidney disease**	237 (30.1)
**Myocardial infarction**	218 (27.7)
**Heart valve disease**	191 (24.3)
**Arrhythmia**	154 (19.6)
**Chronic obstructive pulmonary disease**	123 (15.6)
**Stroke and TIA**	81 (10.3)
**Anemia**	64 (8.1)
**Thyroid abnormality**	48 (6.1)
**Cancer**	17 (2.2)
**History of cigarette smoking**	186 (23.6)
**BMI**	4.24± 26.11*

* Data are presented as mean ±SD; BMI: body mass index; **TIA:** transient ischemic attack

The most important risk factors for the development of HF in these patients were ischemic heart disease (77.9%) and hypertension (63.7%), respectively. Table-2 shows the results of the clinical profile of patients at the time of admission. Most patients (69%) were in class III and IV New York Heart Association (NYHA). Among the registered patients, 74.5% had an EF less than 40%. Half of the patients (49.8%) had peripheral edema, and 64.2% of patients had pulmonary crackles on admission. Troponin level was higher than normal in 8.6% of the patients. Table-3 shows the pharmacotherapy condition before admission and during hospitalization. Among drugs used before hospitalization, antiplatelet had the highest (53.7%) intake and during hospitalization diuretics (84.9%) were mainly prescribed. Table-4 shows the outcome of patients in the follow-up of PROVE/HF. Of the 787 patients registered, 30.9% died over a one-year period. The mean hospital stay was 4.88 days. Also, the re-admission rate for patients with HF was at least once in 16%, among whom 24% had their third re-admission that continued similarly until the fifth to ninth re-admission.

**Table 2:** Clinical Profile and Laboratory Values on Admission

**Variables**	**n(%) or mean±SD**
**Class III and IV of NYHA**	543 (69)
**EF<40%**	586 (74.5)
**Systolic blood pressure (mm Hg)**	127.81 ±26.94
**diastolic blood pressure (mm Hg)**	79.56 ±15.71
**Heart rate**	87.62 ±20.81
**Peripheral edema**	392 (49.8)
**Jugular venous distension**	158 (20.1)
**Pulmonary crackles**	505 (64.2)
**Cold extremities**	24 (3.0)
**Serum sodium (mEq/L)**	139.28 ±5.17
**Potassium (mEq/L)**	4.51 ±0.67
**BUN (mg/dl)**	29.50 ±19.21
**Serum creatinine (mg/dl)**	1.52 ±0.89
Positive troponin*	57 (8.6)

*Troponin was measured qualitatively in some of our centers. Therefore the results are shown either positive or negative. NYHA:New York Heart Association Functional Classification. **EF:** ejection fraction; **BUN:** blood urea nitrogen

**Table 3:** Medications Used Before Admission and During Hospitalization

**Medicines**	**Before admission** **n (%)**	**During hospitalization** **n (%)**
**ACEI**	102 (13)	242 (30.7)
**ARB**	340 (43.2)	431 (54.8)
**ACEI/ARB**	423 (53.7)	608 (77.3)
**BB**	325 (41.3)	482 (61.2)
**Diuretic**	355 (45.1)	668 (84.9)
**Digoxin**	216 (27.4)	401 (51)
**Aldosterone antagonist**	195 (24.8)	422 (53.6)
**Statins**	307 (39)	539 (68.5)
**Intravenous inotrope**	0	106 (13.5)
**Nitrates**	348 (44.2)	573 (72.8)
**Antiplatelet**	423 (53.7)	610 (77.5)
**Warfarin**	152 (19.3)	180 (22.9)

**ACEI:** angiotensin-converting-enzyme inhibitor, **ARB:** angiotensin receptor blockers; **BB:** beta-blockers

**Table 4:** Patients Outcomes in the First Year of Follow-Up

**Outcomes**	**n (%)**
**Total death in the first year of follow-up**	243 (30.9)
**Death in hospital**	49 (6.2)
**Death in first follow** **(1 month)**	80 (10.2)
**Death in second follow** **(6 months)**	69 (8.8)
**Death in third follow (12 months)**	45 (5.7)
**Total live in the first year of follow-up**	474 (60.2)
Unknown*	70 (8.6)
**Total registered patients**	787
**Re-hospitalization within a year**	
**Second hospitalized**	129 (16)
**Third hospitalized**	31 (24)
**Fourth hospitalized**	16 (52)
**Fifth to ninth hospitalized**	12 (75)
**Length of stay (day**	† 4.88 ±3.83

* Patients who for whatever reason were not available for follow-up † Median (Q1, Q3) =4 (3, 6)

## Discussion

PROVE/HF is a young pilot national registry system continually adding new registrations. Since this registry is hospital-based, only patients with acute or exacerbated and decompensated HF were included, so it has not evaluated all patients, such as those patients with chronic HF. Our study reviewed the demographics and primary results of the first year of registration and the overall outcome of the follow-ups.

The mean age of patients was 70.74 years. In most registry studies, the mean age of patients with HF was almost 70 years [18, 20, 24, 27], while, in some registries, such as CARE GULF, the mean age was 59 years, which may be related to the younger population and increased risk of cardiac risk factors in younger ages [28].

The percentage of men and women with HF are different. In our registry, men consist 60.7% of all patients, a finding that is consistent with some studies [18, 25, 26, 28, 29], while others have reported more women [20, 24, 27]. Our finding may be attributed to the fact presented by the available studies in Iran, in terms of higher risk of cardiovascular disease in men than women and that women develop cardiovascular diseases in an older age, compared to men [5]; and the subsequent risk of HF as the final common route of cardiovascular diseases is higher in men.

In examining the medical history, it was revealed that the most common risk factors for the development of HF in these patients were ischemic heart diseases, hypertension, and diabetes mellitus, respectively that is consistent with most registry systems of HF [18-20, 24-30]. The mean BMI of patients with HF was 26.11±4.24, and 60% of patients were overweight or obese. However, since patients’ dry weight is usually not measured, these findings may be affected by edema on admission.

As 74.5% of our patients had an EF less than 40%, showing that most patients with HF had reduced EF, similar to the results of the European and the Middle East registries and was higher compared to American registries [28]. This shows that a higher percentage of PROVE/HF patients had left ventricular systolic dysfunction, expressed as left ventricular EF less than 40%. Of course, sometimes studies have considered a higher cut-off point for EF.

Therefore, if we considered 50% to 40%, the percentage of cases with low EF would be more than 74%. Also, 69% of patients were in NYHA III and IV. As the present registry was hospital-based, patients with functional class deterioration were admitted to the hospital. Most patients had normal blood pressure, and only 3% had symptoms of low peripheral perfusion such as cold extremities. Peripheral edema was also present in only half of the patients. According to the global statistics, less than 5% of patients had symptoms of cardiogenic shock, such as low pressure, cold extremities, and cyanosis [31]; we also observed cold extremities in 3%.

Pulmonary crackles also existed in about 60% of patients; thus, not having pulmonary crackles on auscultation does not rule out the diagnosis of HF as a cause of dyspnea. Relevant literature has also reported its sensitivity to be 15% [32]. The jugular venous pressure was higher than normal in one-fifth of the patients, but the sensitivity of this finding to assess left ventricular filling pressure has been reported to be about 70%. However, a very important factor, in this case, is the apparent inter-observer differences for its correct estimation.

In clinical studies, creatinine levels were slightly higher than normal, which can be due to a higher incidence of kidney failure in patients with HF. Troponin levels were higher than normal in 8% of patients, which could be attributed to causes other than myocardial ischemia, such as kidney failure or pulmonary embolism. In this study, retrospective use of medication was reported before and during hospitalization. Retrospective Medical Use Evaluation (MUE) can be used to identify the shortcomings of prescription and can be useful in future educations and needs assessment [33].

According to the heart failure treatment guidelines [34], angiotensin-converting enzyme (ACE) inhibitor, angiotensin receptor blocker (ARB), and beta-blockers are the cornerstones of treatment of HF, but as presented in Table-3, the percentages of prescribing these drugs for patients who referred to the hospital with decompensated HF were 13%, 43.2%, and 41.3%, respectively, and 53.7% received ARB/ACEI. The percentage of ACEI and ARB drugs before admission was less than most registry studies and like Acute Decompensated Heart Failure Syndromes (ATTEND) [18], ARB was administered more than ACEI. Underutilization of ACEI is perhaps related to reasons such as contraindications, side effects, or active choice [19]. Also, administration of ACEI less than ARB in these patients was unlike Western countries, and guidelines. Therefore, in prescribing main drugs for patients with HF, in addition to the underutilization, there was a failure to comply with guidelines. This is certainly one of the main factors in disease progression, mortality, and readmission rate in these patients.

In PROVE/HF, mean hospitalization was about four days. In Western registries, a range of 4-11 days and in some registers like ATTEND [18], 31 days were reported. Hospitalization period depends on several factors, such as the types of healthcare systems, the incentive for care, differences in patterns of medical practice, budget availability, and administration [35]. The results showed that hospitalization period was relatively low in PROVE/HF. On the other hand, we observed higher mortality in 1-month follow-up after discharge than other follow-up periods, while the re-admission rate was high. So maybe, preventing haste in registering patients and more extended hospital stay would lead to further evaluation of their condition, more successful disease control and treatment, and reduced mortality after discharge or re-admission in a short time after discharge. Proper management of patients in hospitals results in better outcomes.

Of course, re-admission rate, in addition to early discharge before stabilization of the patient, is determined by other factors such as improper administration of medications, insufficient follow-up, lack of patient education on self-care, and lack of social support that are all controllable and preventable [33]. The mortality rate in PROVE/HF was 30.9% that is a high percentage, compared to the available studies documenting HF [19, 24, 26]. These patients were re-admitted up to 9 times a year. Lack of appropriate use of drugs for these patients increases morbidity and mortality after discharge [36].

It seems that health policy-makers should mainly focus on programs to identify and improve factors contributing to inadequate intake of drugs (such as lack of physicians’ awareness of the guidelines, lack of local guidelines, involvement of other doctors than the main physician of the patient, incorrect omission or reduction of dose of medications), as well as factors associated with self-administration of medications, lack of follow-up, lack of patients’ awareness on appropriate use of medications, self-care, and implement strategies to improve them.

Although PROVE/HF was the first registration for patients with HF in Iran and provided information on appropriate admission to collect data from patients, it had some limitations, identification of which will help us better interpret the results.

The primary registration of HF in PROVE was cold; therefore, the obtained data was associated with the accuracy of medical documents and patients’ records. Since this registry was retrospective, confounding factors may have affected the results. Also, the cold nature of medical records caused missing data; thus, we tried to complete the missing information and questionnaires as much as possible in follow-up sessions from patients and their families. However, after one year, registration of this disease would change to EORP/HF and according to the related protocol as hot pilot.

Patients follow-up was performed by telephone and interview with patients and their relatives. Also, the accuracy of data collection was based on drug accuracy, awareness, and other information of the follow-up checklist.

On the other hand, this registry was hospital-based and, of course, the population-based registry will provide comprehensive information about these patients.

## Conclusion

PROVE/HF showed a high annual rate of re-admission and mortality in patients and prescription of drugs inaccordance with the guidelines for the treatment of HF and other registries. Further assessment and policy-making are required to manage patients according to national guidelines that will lead to successful control and treatment.

## Conflict of Interest

All authors declare that there is no any conflict of interest.
